# HOW MUCH IS WORKPLACE FLEXIBILITY WORTH TO OLDER WORKERS? AN ANALYSIS OF MONETARY AND NONMONETARY TRADE-OFFS

**DOI:** 10.1093/geroni/igad104.1148

**Published:** 2023-12-21

**Authors:** Kevin Cahill, Michael Giandrea, Joseph Quinn, Loretta Platts

**Affiliations:** Boston College, Boston, Massachusetts, United States; U.S. Bureau of Labor Statistics, Washington, District of Columbia, United States; Boston College, Boston, Massachusetts, United States; Stockholm University, Stockholm, Sweden

## Abstract

To what extent are older workers willing to forgo wages to obtain a flexible work arrangement when transitioning from career employment later in life? Older workers generally, and pension-eligible workers in particular face a plethora of trade-offs when it comes to continued work later in life. In this paper, we examine the components of older workers’ total compensation and explore how the mix of compensation changes as workers transition out of the labor force. Specifically, we focus on the relationship between monetary compensation (wages, fringe benefits) and workplace flexibility (phased retirement, hours flexibility), and explore the degree to which workers, by their choices, are willing to accept lower wages in return for increased flexibility on their job. To estimate this relationship, we use longitudinal, biennial data on thousands of older Americans representing four different cohorts available from the nationally-representative Health and Retirement Study (HRS). The HRS began in 1992 and, therefore, contains 30 years of follow-up data for the oldest cohort. For each respondent, we construct individual work histories and examine individuals’ evolving wage and hours arrangements from career employment to complete labor force withdrawal. Using multivariate techniques, we find that, all else equal, individuals experience a modest 5.7 percent decline in hourly compensation in return for a 20 percent reduction in hours worked. The findings from this paper can inform policymakers interested in continued work later in life about the degree to which older workers are willing to forgo the financial benefits of work in return for flexible schedules.

